# Plasmid-Based Gene Expression Systems for Lactic Acid Bacteria: A Review

**DOI:** 10.3390/microorganisms10061132

**Published:** 2022-05-31

**Authors:** Tawsif Ahmed Kazi, Aparupa Acharya, Bidhan Chandra Mukhopadhyay, Sukhendu Mandal, Ananta Prasad Arukha, Subhendu Nayak, Swadesh Ranjan Biswas

**Affiliations:** 1 Department of Botany, Visva-Bharati University, Santiniketan 731235, West Bengal, India; prince.evankazi@gmail.com (T.A.K.); aparupa.acharya@gmail.com (A.A.); bidhutntd@gmail.com (B.C.M.); 2 Laboratory of Molecular Bacteriology, Department of Microbiology, University of Calcutta, 35, Ballygunge Circular Road, Kolkata 700019, West Bengal, India; sukhendu1@hotmail.com; 3 Researcher 5 Department of Neurosurgery, Medical School, University of Minnesota, Minneapolis, MN 55455, USA; arukh001@umn.edu; 4 Sr. Scientist, Clorox, Better Health VMS, Durham, NC 27701, USA; subhendu.nayak@clorox.com

**Keywords:** lactic acid bacteria, plasmid-based vectors, NICE system, theta-mode of replication, rolling-circle replication, constitutive expression

## Abstract

Lactic acid bacteria (LAB) play a very vital role in food production, preservation, and as probiotic agents. Some of these species can colonize and survive longer in the gastrointestinal tract (GIT), where their presence is crucially helpful to promote human health. LAB has also been used as a safe and efficient incubator to produce proteins of interest. With the advent of genetic engineering, recombinant LAB have been effectively employed as vectors for delivering therapeutic molecules to mucosal tissues of the oral, nasal, and vaginal tracks and for shuttling therapeutics for diabetes, cancer, viral infections, and several gastrointestinal infections. The most important tool needed to develop genetically engineered LABs to produce proteins of interest is a plasmid-based gene expression system. To date, a handful of constitutive and inducible vectors for LAB have been developed, but their limited availability, host specificity, instability, and low carrying capacity have narrowed their spectrum of applications. The current review discusses the plasmid-based vectors that have been developed so far for LAB; their functionality, potency, and constraints; and further highlights the need for a new, more stable, and effective gene expression platform for LAB.

## 1. Introduction

Lactic acid bacteria represent a family of non-pathogenic, non-sporulating, microaerophilic, Gram-positive bacteria that produce lactic acid as the major end resultant during carbohydrate fermentation and have been employed in food supplements, medicines, and cosmetics for ages to promote modern human civilization [1,2]. LAB are generally recognized as safe (GRAS) and due to this status, these food-grade Gram-positive bacteria have been used as starter cultures for food fermentation and as cell factories for the production of various macromolecules, enzymes, and relevant metabolites in the food, pharmaceutical, and dietary supplement industries [3,4,5,6,7]. These microbes not only support the fermentation process but also improve the organoleptic and rheological properties of food products [8]. Furthermore, due to their ability to produce various bacteriocins, they also help to extend the shelf life of various foods [4,9,10,11,12]. LAB are used as probiotics to promote human health as they maintain and modulate the intestinal flora; prevent hypersensitivity reactions and stimulate the immune response; and help protect against pathogens by producing antibacterial peptides, such as bacteriocin, in the gut [13]. Probiotic lactic acid bacteria are also used to treat several diseases such as diarrhea, inflammatory bowel diseases, and autoimmune diseases [14,15,16,17]. Because of their ability to efficiently produce recombinant proteins, LAB are considered as emerging candidates for the expression of proteins of medical, industrial, and biotechnological relevance [18,19,20]. Recently, *Pediococcus* sp. has progressively become an attractive and promising host for the development of next-generation probiotics and microbial cell factory for potential applications as live delivery vectors for use as therapeutics [13]. In the last decade, scientific interest has focused on the application of this group of food-grade bacteria as effective vehicles for mucosal delivery and for the delivery of recombinant prophylactic and therapeutic proteins, neutralizing antibodies, and the variable domain of heavy-chain-only antibodies from camelids—known as VHH antibodies or nanobodies—into the human gut, to prevent and treat inflammatory bowel diseases (IBDs), autoimmune disorders, infectious and non-infectious gastrointestinal diseases, and infections by pathogenic microorganisms from mucosal surfaces [18,21,22,23,24,25,26]. As lactic acid bacteria (LAB) is a cell factory and delivery vehicle for the delivery of therapeutic proteins, an LAB strain (*Lactococcus lactis*) was used as a vector to deliver HuNoV (human noroviruses) antigen and it demonstrated that this LAB-based HuNoV vaccine induced protective immunity in gnotobiotic piglets [27].

In modern times, advances in genetic engineering and the development of several sophisticated genome engineering tools, such as CRISPR/Cas, RecT-mediated recombineering, etc., have created opportunities to further expand the biotechnological and industrial application of LAB. For the expression and delivery of recombinant proteins and RNA for successful genome engineering using these tools, plasmid-based vectors are essential [28]. Plasmids are extrachromosomal, covalently closed, circular, and self-replicating DNA molecules that are important for the rapid adaptation of bacterial populations to different environmental conditions, protection from phages, niche development, and community structure shifts [11]. Because many LABs have played critical roles in human well-being, from food production to drug delivery, plasmids have been extensively studied to expand the uses of LAB through genetic modification, and hence research continues to develop gene expression systems [20]. This review focuses on the plasmid-based vectors developed for LAB to date, and their functionality, efficacy, and applications. The need for a new, stable, and effective gene expression platform for LAB has also been outlined.

## 2. Components of Plasmid-Based Vectors

Discovered in the early 1950s, plasmids were first conceived as vectors for gene cloning in 1972 when a small group of scientists consisting of Stanley Cohen, Herbert Boyer, and Charles Brinton used the enzyme *EcoRI* to linearize the tetracycline-resistant plasmid pSC101 and cloned a kanamycin-resistant gene to develop double-resistant pSC102. The success of this study not only established pSC101 as the first plasmid-based cloning vector but also revolutionized the concept and development of molecular biology [29,30].

A fundamental precondition for efficient genetic manipulation of organisms is the availability of appropriate vectors that guarantee duplication and maintenance of both the vector DNA and the implanted foreign DNA. For a plasmid to be an efficient expression system, it should have several distinct features: a functional and stable replicon to maintain the recombinant plasmid in the host system; a selectable marker, such as an antibiotic resistance gene or a bacteriocin immunity gene, etc., for the selection of positive recombinants; a strong promoter with an associated ribosome-binding site (RBS) along with other regulatory sequences for an effective inducible or constitutive production of the protein of interest; and a multiple cloning site adjacent to the promoter region for successful in-frame cloning of the desired gene to achieve expression. However, if the vector is constructed for food-grade application, all DNA components must be acquired from food-grade organisms with a GRAS and/or Qualified Safe Acceptance Status (QPS) and no antimicrobial resistance will be entertained as mandated by the US Food and Drug Administration and by the European Food Safety Authority [20,31].

### 2.1. Replicon and Mode of Replication

A replicon, the most important region in the plasmid-based expression system, consists of the origin of replication (*ori*) and all its control elements. The replicon is the site of DNA replication initiation that allows a plasmid to reproduce itself and survive within the host cell. It is generally an A-T-rich sequence where the DNA melts, allowing the replication machinery to step in and actively participate in the making of copies. The host range of a vector depends mostly on the plasmid’s replicon. Not only the host range but also the segregation stability, i.e., the ability of the vector to segregate into daughter cells during cell division, is replicon dependent [32,33]. There are two types of plasmid replication mechanisms, namely the rolling circle or sigma mode of replication (RCR) and theta mode of replication [34,35].

#### 2.1.1. Rolling Circle Replicating Plasmids

Rolling circle replicating (RCR) plasmids [34,36] are omnipresent in both Gram-positive and Gram-negative bacteria and even in archaea [37]. The usual elements of RCR plasmids are the replication initiator (Rep) protein, the double-stranded origin (*dso*), and the single-stranded origin (*sso*). The unidirectional and disengaged synthesis of the primary strand and the lagging strand are the two key phases of the sigma replication mode [34,38,39,40]. Plasmid-encoded Rep protein binds to the cognate *dso*, which consist of either an inverted repeat or a set of two to three direct repeats either adjacent to the nick site or separated by up to 100 bp [34,38,40], and initiates plasmid replication with the introduction of a single-strand nick at the specific nick site. This consists of an inverted repeat, on the parent plus strand, leaving a free 3′-OH, which in turn serves as a primer for leading strand synthesis. Leading strand synthesis generates a single-stranded DNA (ssDNA) replication intermediate [37,40]. Conversion of the displaced ssDNA intermediate to double-stranded DNA (dsDNA) occurs through lagging strand synthesis beginning at the *sso* [34,38,39,40]. There are several families of these plasmids which can be classified as the pT181, pE194/pMV158, pC194, and pSN2 family. In general, RCR-type plasmids from Gram-positive bacteria exhibit a broad host range and high copy number as host dependency is non-existent; hence, they are ideal for vector construction. However, having an ssDNA replication intermediate, they exhibit low structurally and segregationally stability, making them unsuitable for the construction of expression systems for starter cultures and drug delivery systems [32,34,37,40]. RCR plasmids are highly disseminated, so they possess the threat of spreading the desired trait or resistance to the undesired organisms, which could affect food production or vaccine delivery.

#### 2.1.2. Theta-Type-Replicating Plasmids

The marginal replicon of a theta-replicating plasmid consists of the *ori* region and a gene encoding a replication initiator protein. The *ori* region is an AT-rich region usually followed by iterons (three and a half 22 bp direct repeats) and two tiny, inverted repeats that overlap the -35 element of the *rep* promoter and the RBS of the *rep* gene. Theta plasmid replication is initiated with the softening of the parental strands, followed by the continuous and discontinuous production of the leading and lagging strands, respectively [34]. The iterons of the *ori* site are not only crucial for the initiation of replication [34,41] but also important for the control of plasmid replication [34,41,42,43]. To date, six classes of theta replicons [41,44] have been identified, class A-F: Class A includes plasmids that have a Rep protein with a related origin of replication and replicate independently of DNA Pol I. Most LAB theta-type-replicating plasmids belong to class A. Theta replicons of plasmids, such as pCI305 [45], pUCL22 [46], pWVO2 [47], pW563 [48], pCD4 [44], pSM409 [49], etc., from lactic acid bacteria are representatives of class A theta replicons. Class B replicons lack *oriA* and do not encode a Rep protein. Their replication is initiated by processing a transcript synthesized by the host RNA polymerase. Interestingly, class C replicons do not have an *oriA*-like structure but encode a replication protein and require DNA Pol I for replication. pColE2 and pColE3 are the most known class C replicons. Class D replicons, although dependent on the plasmid-encoded replication protein (Rep), is independent of the DNA structure typical of the origin of replication of most Rep-dependent plasmids, and is initiated by DNA polymerase I. Some LAB theta-type-replicating plasmids (pAMβl, pEF1, pIP501, and pSM19035) belongs to this class. Class E replicons are yet to be researched and characterized and are represented by plasmid pLS20 from *Bacillus subtilis*. As observed, the structural organization of the minimal replicon of pLS20 differs from the typical plasmids of Gram-positive bacteria. Class F replicons possess a replication initiation protein (RepN) but lack the characteristic AT-rich region at the replication initiation site. The origin of replication differs structurally from those of class A and is identified by the presence of multiple iterons on the coding sequence of RepN, but replication is independent of DNA polymerase I. Plasmids of diverse origins, such as pLJ1 of *Lactobacillus sp.* [50], pAD1 [51], pCF10 [52], and pPD1 [53] from *Enterococcus* sp., and the plasmids pNP40 [54] and pLS32 from *B. natto* and pCI2000 [55] from *Lactococcus* sp. belong to Class F.

The creation of theta plasmids can be uni- or bi-directional and can initiate from multiple origins, and produces double-stranded DNA replication intermediates compared to the RCR plasmid; therefore, theta-replicating plasmids show structural and segregational stability compared to RCR plasmids and can incorporate and maintain large DNA fragments, making them useful as cloning and expression vectors [32,56].

### 2.2. Selectable Marker

The selectable marker aids in the identification and elimination of non-transformants and selectively allows transformants to grow in the presence of appropriate selective pressure. A selectable marker for a vector is, therefore, mandatory for efficient screening and easy handling [20,33,56]. In general, antibiotic resistance genes are considered to be the most suitable marker for the vector, since they can specifically eliminate the untransformed cells and the transformants can only survive in the presence of suitable antibiotics. Apart from antibiotics, bacteriocin production and immunity, phage resistance, or heavy metal resistance have been identified as efficient and straightforward mechanisms to separate the transformants from the non-transformed ones and are, therefore, also used by researchers as markers for vector construction. Since the use of antibiotic resistance genes as selection markers for food-grade vectors has been restricted by regulatory bodies, the sugar utilization gene, bacteriophage resistance, stress tolerance, bacteriocin resistance, and immunity, etc. have been used as selection markers for the development of food gene expression systems for LAB [57].

### 2.3. Promoter

The efficiency of an expression system is often determined by the strength of its promoter and the way it works since the expression of the heterologous proteins is controlled solely by the promoter system [20,56,58,59]. Although several gene expression systems have been developed in LAB, the choice of an appropriate promoter still remains a very important consideration when constructing expression cassettes. For laboratory protein production, the type of promoter (inducible or constitutive) is not a major concern, but when expression for therapeutic applications is desired, several factors come into play [59]. The strong inducible promoters are used to overproduce recombinant proteins of medical and industrial relevance in LAB, whereas the constitutive promoter is preferred for in vivo delivery and steady-state production of therapeutic proteins in the human gut since constitutive promoters do not require external induction, unlike the inducible promoter.

## 3. Challenges in Vector Engineering

When designing a vector, researchers must address several bottlenecks that determine the efficiency of a gene expression system, as follows:

### 3.1. Copy Number

Plasmid-based vectors are self-replicating DNA molecules and maintain a specific number of copies in the host cell [43]. It is believed that a high copy number of a vector supports plasmid segregation into daughter cells during cell division and that an additional burden on the partition system is not required to increase the stability of plasmid segregation; however, it places a metabolic burden on the host cell, which in turn affects the cell’s metabolism [33]. On the contrary, low-copy-number plasmid vectors, while not subjected to host cell metabolic stress, have a high risk of segregationally being unstable [60]. In order to facilitate the process of plasmid segregation, partition systems are often present in low-copy plasmids [53]; however, the presence of partition systems in the vectors is not recommended as it increases the size of the vector, which in turn decreases the vector’s carrying capacity. In addition, the replicon type also plays a crucial role in the copy number and segregational stability, since theta-replicating plasmids mostly have a low to medium copy number and are segregationally stable while rolling circle plasmids have a high copy number and are unstable due to their single-strand intermediate [56]. In fact, gene expression in low-copy vectors has been found to have improved functionality and stability compared to multi-copy plasmids [61]. Therefore, special care should be taken in selecting appropriate replicons for gene expression system design.

### 3.2. Plasmid Incompatibility

In recent years, the use of multiple gene expression systems to express multiple genes simultaneously in a single host in a modular manner has attracted much interest from researchers. However, bacterial plasmids exhibit an idiosyncratic property of incompatibility, meaning that plasmids with similar replication machinery cannot be stably maintained simultaneously in the same host [44,62]. Therefore, it is important to consider plasmid incompatibility while designing new genetic tools for heterologous protein production.

### 3.3. Plasmid Structural and Segregation Stability

Plasmid instability is one of the main problems in research; it also affects pilot-scale fermentation in industry. Researchers have repeatedly reported that plasmids are simultaneously lost during cell division (segregational instability), or DNA segments are rearranged and lost from the plasmid backbone (structural stability) [32,60,63,64]. Segregational instability occurs mainly due to defects in the partition machinery and depends mainly on the plasmid replicon. Therefore, proper selection of the replicon is critical for vector construction. Whereas, when excess sequences homologous to the genome are present in the plasmid backbone, recombination and structural instability of the vector can result. When designing new gene expression constructs, attention must, therefore, be paid to the stability of the vector.

### 3.4. Vector Size and Carrying Capacity

The carrying capacity of a vector is defined as the size of an insert that can be cloned into a gene expression system without affecting the stability and functionality of the plasmid. The vector size plays a crucial role in determining the carrying capacity of the vector since a replicon of a plasmid possesses the ability to carry out the replication of a certain length of DNA; if this limit is exceeded, severe instability is observed as a result of a failure to reproduce. Therefore, before constructing a plasmid-based vector, it is important to characterize the components well (as indicated above) and minimize the junk DNA from the vector backbone to limit the vector size so that a large amount of genetic load can be stably inserted.

### 3.5. Host Range

The host range is one of the most important determinants of vector fitness. A vector’s ability to be stable across a broad host range increases its applicability in modern research, as narrow-host-range platforms are highly specific, only addressing the specific requirements of a particular host, and thereby limiting their use to heterologous hosts [65]. While broad host systems allow for the cloning and expression of genes in a variety of heterologous hosts, researchers can switch to other models without changing the circuitry. The vector replicon primarily determines the host range of the system; therefore, it is important to select a replicon with a broad host range for the development of the gene expression system.

Considering the need for an efficient gene expression system for LAB to widen the spectrum of applications of LAB from the laboratory to the land, the well-characterized components, namely the origin of replication, from food-grade LAB plasmids should be the first choice to construct a vector. Since the origin of replication plays a crucial role in controlling the copy number, stability, host range, incompatibility, and carrying capacity, special care should be taken in the appropriate selection of *ori* for the construction of a stable gene expression system for LAB. Furthermore, as previously mentioned, constitutive expression in LAB is preferred for steady-state overproduction of recombinant proteins of medical and industrial relevance; however, constitutive expression often results in a metabolic burden on the host, which in turn leads to a plasmid instability, followed by plasmid loss. Therefore, a stable theta-type replicon is preferred over a rolling circle replicon for LAB (vide supra).

## 4. Vectors for Lactic Acid Bacteria

There are mainly two types of plasmid vectors: cloning and expression vectors. Cloning vectors are generally used to construct a library to obtain specific DNA fragments of interest for various purposes in molecular biology, such as probe generation, restriction mapping, sequencing, etc., while the expression vectors with their strong transcriptional and translational signal sequences are used to produce various homologous and heterologous proteins in large quantities. Over the past two decades, researchers have been involved in the growth of an array of cloning and expression vectors for the engineering of various LAB species [66]. The vectors developed for LAB or other Gram-positive bacteria that are currently in use are constructed largely based on the previously characterized plasmids of LAB (Table 1) and can be divided into two major classes based on the replicon used, which are as follows:

Class 1 vectors: These vectors are represented by the large theta-replicating, segregational and structurally stable conjugative plasmids pIP501 and pAMβ1 and their derivatives, which are resistant to macrolides, lincosamides and spectogramin B (MLS resistance), and other antibiotics [53,67,68,69]. These plasmids have a broad host range and can replicate in various LAB such as *Lactococcus* spp., *Lactobacillus* spp., and *Pediococcus* spp. [70,71,72]. However, pIP501 and pAMβ1 derived from streptococci and enterococci cannot be considered food-grade since these organisms do not have GRAS status, and are, therefore, not suitable for food and pharmaceutical applications.

Class 2 vectors: These vectors are derived from small cryptic plasmids (pSH71, pD125, pCL2.1, pWC1, pBM02, and pWV01) of several lactococcal species. Being rolling circle plasmids, these vectors are generally segregationally and structurally unstable and have a very low carrying capacity; they are only useful for cloning smaller genes. At the same time, however, pSH71 and pWV01 replicons and their derivatives have a broad host range [73,74].

In addition to these classes of vectors, research is driven towards the development of stable gene expression systems derived from plasmids of food-grade LAB [66]. Expression systems for LAB can be broadly classified into two major types:

### 4.1. Constitutive Gene Expression Systems

As mentioned above, constitutive gene expression systems for LAB have a wide range of biotechnological applications in the development of next-generation health-promoting bio-functional foods and LAB-based therapeutics. To date, a variety of constitutive gene expression systems for LAB have been developed, particularly for *Lactococcus* sp. and *Lactobacillus* sp., using the promoters from housekeeping genes [31,75]. A constitutive gene expression system pUBU was engineered using the theta-type replicon of pUCL287 from *Tetragenococcus halophilus*, the promoter of the L-lactate dehydrogenase (*ldh*L*)* gene from *Lactobacillus plantarum*, and the cadmium resistance (Cd^r^) gene from pND918. The host range of this vector protracted up to *Lactobacillus*, *Lactococcus*, *Pediococcus*, *Enterococcus*, *Leuconostoc*, and *Tetragenococcus* and was able to remain stable for at least 100 generations under non-selective pressure [76]. A constitutive expression system comprising a constitutive L-lactate dehydrogenase promoter (PldhL) from *L. sakei* and a gene downstream of the promoter encoding green fluorescent protein (GFP) has been reported [77]. A constitutive gene expression cassette, pTRK892, was recently developed for *Lactobacillus* sp. The vector was generated using the *Lactobacillus acidophilus* phosphoglycerate mutase promoter (Ppgm) and the pWV01 replicon and the gene cloned into this vector was found to be constitutively expressed at a level that was more than 10-fold higher than that observed in inducible vectors [78]. Recently, pPBT-GFP, a constitutive gene expression system based on the erythromycin (ery) gene promoter, was developed. The low copy number shuttle vector pPBT-GFP consists of pCP289 and pLES003 replicons and is capable of stably replicating in host cells in multiple hosts and can be used as an ideal tool for improving LAB strains of commercial value using genetic engineering [79].

Despite the immense importance of constitutive gene expression systems for lactic acid bacteria in industrial settings, their development has been highly retarded due to the scarcity of constitutive promoters characterized in LAB [59,80]. To date, a handful of constitutive promoters of LAB have been characterized, including P8: Phosphopentomutase promoter; P5: Hypothetical protein L1010 promoter; P6: Hypothetical protein L141485 promoter; etc. [59]. However, these were mostly conditionally constitutive, i.e., they are constitutively active in the presence of certain sugars, and the expression of these promoters was generally weak. The commercially available constitutive vectors pNZ2103 and pNZ2122 from Mobitec are constructed using the *lacA* promoter from *Lactococcus lactis* [81]. The *lacA* promoter of *L. lactis* is believed to be regulated by *lacR* (repressor) and in the presence of glucose, the promoter is inactivated. The expression of the *lacA* promoter was reported to be constitutive in particular hosts. In *L. lactis* MG1363, the activity of the promoter was quite high in the presence of glucose. Whereas, in another host *L. Lactis* MG5267 (*lacR*^+^), the expression of the *lacA* promoter was greatly reduced in glucose and the promoter was induced in the presence of lactose [81]. Therefore, the *L. lactis lacA* promoter is not a constitutive promoter in the strict sense, and thus, the constitutive expression of this vector is host specific.

### 4.2. Inducible Expression Systems

Inducible systems for LAB have received considerable attention worldwide for the overproduction of various proteins in lactic acid bacteria. To date, many inducible gene expression systems exist and are employed extensively in laboratory and industrial settings. Recently, a series of sugar-inducible expression systems pTRK888 (Pfos), pTRK889 (Plac), and pTRK890 (Ptre) were constructed for use in lactobacilli, based on the broad host range replicon of pWV01 and the use of promoters from FOS (Pfos), lac (Plac), and tre (Ptre) operons [78]. In addition, thermo-inducible expression systems and stress-inducible expression systems have also been developed, which Landette discussed extensively in 2017 [20]. Recently, pMY01 was developed using the PsrfA promoter to fine-tune gene expression in a broad range of LAB hosts, namely *L. casei* 5257, *L. plantarum* 97, *L. fermentum* 087, and *Weissella confusa* 10, yielding the recombinant strain *L. casei* 5257-01, *L. plantarum* 97-01, *L. fermentum* 087-01, and *Weissella confusa* 10-0. In addition, the promoter PsrfA was used to construct an autoinducible expression system in *B. subtilis* and *E. coli* [82]. Among the inducible systems, the two-component induction system of the nisin operon has gained relatively more attention due to its stringency of induction and expression and thus a myriads of gene expression systems, the nisin-controlled expression system (NICE), have been developed based on the nisin promoter (PnisA) from *Lactococcus lactis*.

Nisin is a bacteriocin generated by certain strains of *Lactococcus lactis*. It is known that a cluster of eleven genes transcriptionally organized into four operons is involved in the biosynthesis of mature nisin following post-translational modifications. Molecular characterization of the *nisA* promoter revealed that the promoter is self-managed by a two-component regulatory system, comprising the sensor kinase NisK and the response regulator NisR, that responds to extracellular nisin [83,84,85]. Since the nisin-mediated autoinduction of PnisA occurs only in the nisin-producing *L. lactis* via NisR and NisK, a host-vector system was developed by exploiting the auto-induction mechanism of the nisin operon through the chromosomal insertion of the genes involved in signal transduction, *nisK* and *nisR* into *L. lactis* subsp. *cremoris* MG1363 (nisin-negative), creating the strain NZ9000 [84,85]. Likewise, several genetically engineered hosts have been developed to utilize this auto-induction mechanism to produce heterologous proteins in LAB (Table 2). In addition, plasmids have been constructed to facilitate translational and transcriptional fusions and intracellular fabrication or secretion of the gene product.

The vector pNZ8048 is mostly employed for translational fusions, where a gene of interest can be inserted at the canonical *NcoI* site around the ATG start codon for expression. At the same time, two further variants of this plasmid were constructed: pNZ8148 and pNZ8150, wherein pNZ8148, a 60-bp remnant DNA-fragment of *Bacillus subtilis*, was deleted, and in plasmid pNZ8150, the *NcoI* site was swapped for a *ScaI* site that is present just upstream of the ATG start codon. The upgraded pNZ8148 eschews the drawback created by the mandatory usage of the *NcoI* site, which sometimes mandates changing the first base of the second codon in the gene being pursued. In pNZ8150, the pursued gene may be amplified, beginning at the ATG start codon and straightforwardly fused to the vector, resulting in an accurate *nisA* translational fusion [85]. Although several nisin-inducible systems have been developed for efficient gene expression in the engineered host, this host specificity has limited the applicability of these vectors for the genetic engineering of industrially important LAB. Therefore, other strategies were used for a wide range of applications, and a vector pMSP3535 was developed that carries both the *nisRK* genes and the *nisA* promoter on one plasmid, facilitating the induction of gene expression by exogenous addition of nisin [86].

A food-grade-inducible gene expression system has also been created for the expression of heterologous proteins in *L. lactis* [87]. This system consists of a food-grade cytoplasmic-inducible expression vector pRNA48 containing the α-galactosidase gene, theta replicon from pRAF800, and PnisA-MCS-TpepN from pNZ8048; and a cell-wall-anchored expression vector pRNV48 containing α-aga, theta replicon, and PnisA-SPUsp45-nucA-CWAM6-t1t2 from pRNA48 and pVE5524 have been described. The OprF/H fusion derived from *Pseudomonas aeruginosa* was cloned into plasmids pRNA48 and pRNV48 to construct the pRNA48-OprF/H and pRNV48-OprF/H for the expression of OprF/H using a nisin-inducible system, resulting in 9.6% intracellular soluble protein and 9.8% cell-wall-anchored protein in *L. lactis* NZ9000, respectively [87]. Along with nisin-inducible systems, a series of vectors, the pSIP series, was developed using a regulatable promoter involved in the production of the bacteriocins sakacin A and sakacin P, and the genes encoding the cognate histidine protein kinase and response regulator that are necessary to activate this promoter upon induction by a peptide pheromone. The pSIP series of vectors has been used for tightly controlled and efficient expression of β-glucuronidase in both *L. sakei* and *L. plantarum* [88]. A *Lactobacillus/Escherichia coli* shuttle vector, pKRV3, was constructed, including the sakacin signal transducing system. The *gusA* gene fused to the PsapA promoter cloned into this vector allowed for inducible beta-glucuronidase expression in *L. sakei* and *L. plantarum* after induction with sakacin A [89]. A bile-responsive *E. coli*-*Lactobacillus* shuttle expression vector, pULP3-P_LDH_, for *Lactobacillus* spp. was generated by fusing pUC19 with the *L. plantarum* plasmid pLP27, which has been found to persist in *L. plantarum* for up to 80 generations without selection pressure [90].

## 5. Constraints of Gene Expression in LAB

Plasmid-based vectors for LAB are an important tool for large-scale protein production and for the introduction of new traits aimed at improving nutrition and health [91,92]. Due to the ease of recombinant protein production in LAB, many heterologous proteins have been overexpressed in LAB in laboratories and industries for food, pharmaceutical, and nutraceutical applications (Table 3).

However, in most cases, the proteins are overproduced in *E. coli* or *Saccharomyces cerevisiae*, purified, and used directly in the pharmaceutical industry [33,115,116] while in the food industry proteins, vitamins, folic acid, acetaldehyde, and diacetyl are processed in the LAB with inducible systems and administered via food after purification [117,118]. The expression and purification of proteins for food-grade applications is quite a difficult and costly task, so researchers have focused on the development of efficient constitutive gene expression systems to prepare the protein of interest for hassle-free implementation in the food, pharmaceutical, and dietary supplement industries [59,119,120,121,122]. Furthermore, vectors are essential not only for the production of metabolites in the food system for direct administration without purification but also for exploring the very fundamental and unexplored areas of LAB research.

LAB is an extremely important group of food-grade bacteria that has attracted a lot of attention from scientists around the world. Their practical application through genetic engineering is limited due to the lack of effective gene expression systems. Although a handful of reports are available for the construction of the LAB gene expression system, only one set of vectors, MobiTec’s pNZ series, is commercially available. The pNZ series of vectors are mostly NICE systems (nisin-inducible controlled gene expression) in which the production of heterologous proteins is driven by a nisin-inducible promoter. In addition to the inducible gene expression platforms, the pNZ vector series also offers the constitutive systems (pNZ2103 and pNZ2122) for the inducer-free production of proteins. As indicated, expression of this vector is host dependent, *i.e.*, in *L. lactis* NZ3000 (∆*lacF* mutant of *L. lactis* MG5267). The pNZ series of vectors are rolling circle replicating plasmids, and in general, their single-stranded intermediate contributes to their plasmid segregational instability during replication and they have a very low carrying capacity. However, due to the scarcity of expression systems, scientists are forced to rely on pNZ vectors.

## 6. Conclusions

Lactic acid bacteria are one of the most common organisms in modern biology, with multiple applications in the food, pharmaceutical, and nutraceutical industries. The huge importance of this group of bacteria has sparked the attention of researchers in developing next-generation LAB starter cultures to widen the spectrum of applications of this group of bacteria. However, due to the lack of appropriate gene expression platforms for LAB, its applications are limited. To overcome this limitation, the development of new gene expression tools for this group of bacteria must be emphasized, considering two essential features, namely, universality and modularity. The adaptability and modularity of a vector would benefit not only fundamental research but also industrial applications, allowing us to enter a whole new era of synthetic biology. To do so, we require a thorough and comprehensive understanding and characterization of all components of a gene expression system to enable us to develop an efficient gene expression system for LAB.

## Figures and Tables

**Table 1:** Replicons used for the construction of vectors for LAB.

Origin of Replication	Organism	Replication Type	Reference
pWV01	*L. lactis*	Rolling circle	[56]
pSH71	*L. lactis*	Rolling circle	
pCI305	*L. lactis*	Theta	
pAM*β*1	*L. latcis*	Theta	

**Table 2:** Host and vectors engineered for the development of the NICE system.

**Hosts**	**Properties**	**Reference**
NZ9700	Developed from conjugation between MG1614 (RifR StrpR derivative of MG1363) and nisin producer strain NIZO B8. Carries Tn5276 (nisin–sucrose transposon). Frequently used as a nisin producer strain for induction experiments.	[85]
NZ9800	Engineered from NZ9700 with a 4-bp deletion in the *nisA* gene, resulting in inactivation of the nisin operon, with the exception of the *nisRK* genes, which are transcribed from a constitutive promoter. Host of the NICE system.	
NZ9000	*nisRK* gene is integrated into the *pepN* gene of *L. lactis* MG1363 using plasmid pNZ9573. Most frequently used host of the NICE system. *nisRK* integration leads to a pepN-negative phenotype.	
NZ3000	Derivative of the strain MG5267 with a partial deletion of the *lacF* gene rendering the strain unable to grow on lactose. Growth on lactose can be restored by complementing the *lacF* gene via plasmid.	
NZ3900	Derivative of NZ3000; *nisRK* integrated into the pepN gene for the use of the NICE system. Food-grade selection of the transformants can be achieved based on the ability to grow on lactose.	
NZ9000-*htrA*	NZ9000 with complete deletion of the *htrA* gene. Generally used as a host for protein secretion	
**Vectors**	**Properties**	**Reference**
pNZ9573	Plasmid for *nisRK* integration into the *pepN* locus of *L. lactis* subsp. *cremoris*; CmR, EmR.	[85]
pNZ9520	*nisRK* genes under the control of the *rep* promoter of the high-copy-number plasmid pIL253; EryR	
pNZ9530	*nisRK* genes under the control of the *rep* promoter of the low-copy-number plasmid pIL252; EryR.	
pNZ8048	Standard vector generally used for translational fusion at the *NcoI* site; CmR	
pNZ8148	Derived from pNZ8048 by a 60 bp deletion of the residual DNA from *Bacillus subtilis.* Standard vector generally used for translational fusion at the *NcoI* site; CmR.	
pNZ8150	Standard vector generally used for translational fusion at the *ScaI* site; CmR.	
pNZ8021	Vector for transcriptional fusions; CmR	
pNZ8110	Standard vector used for protein secretion using the *L. lactis* Usp45 signal sequence, translational fusion at the *NaeI* site; CmR.	
pNZ8008	Promoterless *gusA* gene under the control of the nisin promoter, used for study of the nisin-controlled expression in *L. lactis* and other hosts; CmR.	
pMSP3535	Vector contains both *nisRK* and PnisA for autoinduction of the NICE system, pAMβ1 and ColE1 replicons; EryR.	

**Table 3:** Heterologous proteins produced in LAB using the plasmid-based gene expression system.

Vector Used	Host Strain	Promoter	Product	Reference
pNZ8048	*L. lactis* NZ9000	Pnis	Intracellular rotavirus spike-protein subunit VP8	[93]
pNZ8048	*L. lactis* NZ9000	P45 series	*nisZ*, *ermC*	[59]
pNZ8048	*L. lactis* NZ9000	PczcD	GFP	[94]
pNZ8048	*L. lactis* NZ9000	Pnis	*GroESL*	[95]
pNZ8148	*L. lactis* NZ9000	Pnis	Heme oxygenase-1	[96]
pNZ8048	*L. lactis*	Pnis	*α*-amylase/*S. aureus* nuclease	[97]
pNZ8148	*L. lactis* NZ9000	Pnis	Pentadecapeptide BPC-157	[98]
pNZ8148	*L. lactis* NZ9000	Pnis	Albumin binding domains	[99]
pNZ8148	*L. lactis* NZ9000	Pnis	p19-Thioredoxin fusion protein	[100]
pNZ273	*L. lactis* NZ9000	Pnis	*β*-glucuronidase (*gusN*)	[84]
pOTHY12	*L. lactis* MG1363	PthyA	Interleukin 10 (IL-10)	[56]
pRV300	*L. lactis* NZ9000	Pnis	*nucB*	[101]
pDK6	*L. lactis* MG1363, *L. lactis* NZ9000	Pnis	Elafin, Secretory Leukocyte Protease Inhibitor (SLPI)	[102]
pSEC	*L. lactis* NZ9000	Pnis	Pancreatitis-associated protein I (PAP)	[103]
pFUN	*L. lactis* MG1363	Pzn	*uspnuc*	[104]
pAK80	*L. lactis* MG1363	P170	*orfX* protein	[105]
pLB141	*L. lactis* MG1363	PgroESL	*nucB*	[106]
pGEM	*L. lactis* NCDO2118, *L. lactis* IL1403, *L. lactis* MG1363, *L. lactis* NZ9000	PxylT	*nucB*	[107]
pValac	*L. lactis* NZ9000	PxylT	*β*-lactoglobulin	[108]
pLB85	*L. lactis* MG1363, *L. lactis* KF147	PSPL	Ethanol	[109,110]
pSH71	*L. salivarius* ATCC 11741	Pnis	IL-17, IL-23	[111]
pT1NX	*L. lactis* MG1363	P1	IL-27	[112]
pAF100	*Lb. paracasei*	Papf	Antibody fragment	[113]
pDL	*L. lactis MG1363* PH3960	Pnis	Protective *Leishmania* antigen (LACK), IL12	[114]
pSIP	*L. planatarum* NC8, *L. sakei* Lb790	PSPL	*β*-glucuronidase (*gusN*), Aminopeptidase N (*pepN*)	[80]

## Data Availability

All data presented here are publicly available.
